# 4-Ethyl-3-(2-thienylmeth­yl)-Δ^2^-1,2,4-triazoline-5-thione

**DOI:** 10.1107/S1600536809000440

**Published:** 2009-01-10

**Authors:** Monika Wujec, Liliana Mazur, Zofia Rzączyńska

**Affiliations:** a Department of Organic Chemistry, Faculty of Pharmacy, Medical University, 20081 Lublin, Poland; b Department of General and Coordination Chemistry, Faculty of Chemistry, Maria Curie-Skłodowska University, 20031 Lublin, Poland

## Abstract

The title compound, C_9_H_11_N_3_S_2_, exists in the thione form in the crystal structure. The central triazole ring is almost perpendicular to the thio­phene ring which is disordered over two orientations [dihedral angles of 88.5 (7) and 85.7 (8)° for the two orientations]. The crystal structure is stabilized by strong inter­molecular N—H⋯S hydrogen bonds, forming centrosymmetric dimers, and by some weak C—H⋯S inter­actions.

## Related literature

For background on the applications of 1,2,4-triazole and its derivatives, see: Ünver *et al.* (2006 ▶); Dobosz *et al.* (2002 ▶); Jian *et al.* (2005 ▶); Maliszewska-Guz *et al.* (2005 ▶); Al-Soud *et al.* (2004 ▶); Amir & Shikha (2004 ▶); Collin *et al.* (2003 ▶); Demirayak *et al.* (2000 ▶); Palaska *et al.* (2002 ▶); Shivarama *et al.* (2006 ▶). For details of the synthesis, see: Wujec *et al.* (2004 ▶, 2007 ▶). For related structures, see: Yilmaz *et al.* (2005 ▶).
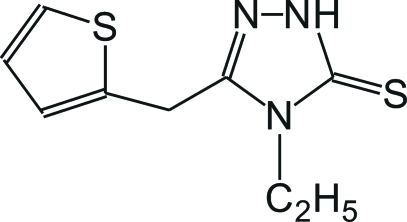


## Experimental

### 

#### Crystal data

C_9_H_11_N_3_S_2_
*M* *_r_* = 225.33
Monoclinic, 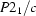

*a* = 6.813 (1) Å
*b* = 17.119 (2) Å
*c* = 9.846 (1) Å
β = 100.88 (1)°
*V* = 1127.7 (2) Å^3^
*Z* = 4
Mo *K*α radiation
μ = 0.44 mm^−1^
*T* = 295 (2) K
0.47 × 0.30 × 0.16 mm

#### Data collection

Oxford Diffraction Xcalibur diffractometer
Absorption correction: none
2735 measured reflections
2592 independent reflections
1130 reflections with *I* > 2σ(*I*)
*R*_int_ = 0.026

#### Refinement

*R*[*F* ^2^ > 2σ(*F* ^2^)] = 0.052
*wR*(*F* ^2^) = 0.111
*S* = 0.98
2592 reflections
147 parameters
H-atom parameters constrained
Δρ_max_ = 0.16 e Å^−3^
Δρ_min_ = −0.17 e Å^−3^

### 

Data collection: *CrysAlis CCD* (Oxford Diffraction, 2005 ▶); cell refinement: *CrysAlis RED* (Oxford Diffraction, 2005 ▶); data reduction: *CrysAlis RED*; program(s) used to solve structure: *SHELXS97* (Sheldrick, 2008 ▶); program(s) used to refine structure: *SHELXL97* (Sheldrick, 2008 ▶); molecular graphics: *SHELXTL/PC* (Sheldrick, 2008 ▶); software used to prepare material for publication: *SHELXL97* and *enCIFer* (Allen *et al.*, 2004 ▶).

## Supplementary Material

Crystal structure: contains datablocks global, I. DOI: 10.1107/S1600536809000440/at2696sup1.cif

Structure factors: contains datablocks I. DOI: 10.1107/S1600536809000440/at2696Isup2.hkl

Additional supplementary materials:  crystallographic information; 3D view; checkCIF report

## Figures and Tables

**Table 1:** Hydrogen-bond geometry (Å, °)

*D*—H⋯*A*	*D*—H	H⋯*A*	*D*⋯*A*	*D*—H⋯*A*
N1—H1⋯S1^i^	0.86	2.44	3.287 (3)	169
C6—H6a⋯S1^ii^	0.97	2.99	3.949 (4)	172
C9—H9⋯S1^iii^	0.93	2.97	3.659 (4)	132
C8′—H8′⋯S2^iv^	0.93	3.02	3.928 (7)	166

Symmetry codes: (i) 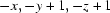; (ii) 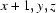; (iii) 
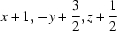
; (iv) 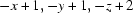.

**Fig. 1.**
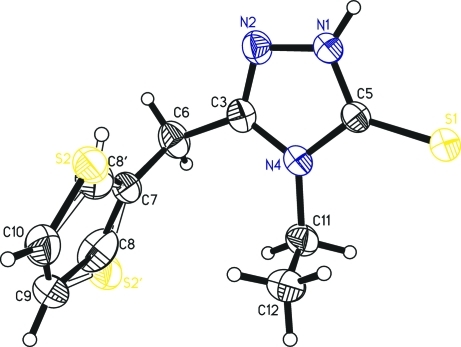
The molecular structure of (I), showing the atom-labelling scheme. Displacement ellipsoids are drawn at the 30% probability level. Both disordered components are shown.

**Fig. 2.**
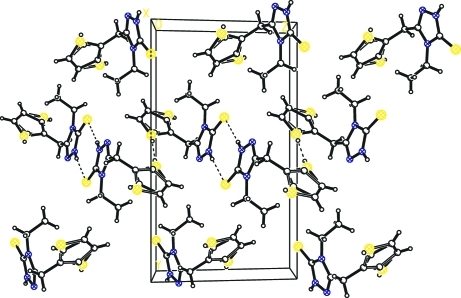
The molecular packing of (I), viewed down the a axis. Dashed lines indicate hydrogen bonds.

## supplementary crystallographic information

### Comment

1,2,4-Triazole and its derivatives represent one of the most biologically active classes of compounds possessing a wide spectrum of activities, such as antimicrobial, fungicidal, anti-inflammatory, antiviral, antitumor or analgesic activity (Al-Soud *et al.*, 2004; Amir & Shikha, 2004; Collin *et al.*, 2003; Demirayak *et al.*, 2000; Maliszewska-Guz *et al.*, 2005; Palaska *et al.*, 2002; Shivarama *et al.*, 2006; Wujec *et al.* 2007). The 1,2,4-triazole nucleus has been incorporated into a wide variety of therapeutically important drugs *e.g.* Fluconazole, Itraconazole, Anastrazole, Ribavirin. In recent years 1,2,4-triazole finds an important place in medicinal chemistry as material for the preparation of antibacterial agents (Demirayak *et al.*, 2000). In this context, we described the synthesis and antibacterial activity of a series of 1,2,4-triazoline-5-thione derivatives (Wujec *et al.* 2004). In the present paper we report the structure of one of them: 4-ethyl-3-(thiophene-2-yl-methyl)-Δ^2^-1,2,4-triazoline-5-thione (I). This compound inhibite the growth of Trichophyton spp.

In the title compound (Fig. 1), the C5—S1 bond length [1.673 (2) Å] is within the values observed for a C=S double bond. In the planar 1,2,4-triazole ring the C3=N2 bond is clearly double, being much shorter then the other C—N bonds. This distance is also comparable to literature data (Yilmaz *et al.*, 2005). The thiophene ring is disordered over two orientatians with respect to the C6—C7 bond; the dihedral angles between the triazole and the thiophene rings for the two orientations of the second one are 88.5 (7) and 85.7 (8)°. Atoms C6 and C11 lie in the plane of triazole, whereas the ethyl atom C12 is signifficantly displaced from the plane of central system as indicate from the torsion angle C5—N4—C11—C12, being of 83.3 (3)°.

The crystal structure is stabilized by strong intermolecular N1—H1···S1 hydrogen bonds, forming centrosymmetric dimers (Fig. 2), together with some weak C—H···S interactions (Table 1).

### Experimental

4-Ethyl-3-(thiophene-2-yl-methyl)-Δ^2^-1,2,4-triazoline-5-thione was synthesized according to the method which was described in a previous paper (Wujec *et al.*, 2004). Prism-shaped colourless single crystals, suitable for X-ray diffraction measurements, were obtained by the slow evaporation of a 2-propanol solution of the compound.

### Refinement

All H atoms were positioned geometrically and allowed to ride on their parent atoms, with N1—H1 distance of 0.86Å and C—H bond distances in the range 0.93 - 0.97 Å. The displacement parameters of the H atoms were *U*_iso_(H) = 1.2 *U*_eq_(C/N). The thiophene ring is disordered over two positions related by a 180° rotation around the C6—C7 bond. This disorder gives rise to two positions for each of the S2 and C8 atoms; the refinement of their occupancies showed that one of these positions is predominant, with an occupancy of 0.538 (4) for S2 and C8 atoms [the other one is with an occupancy of 0.462 (6) for S2' and C8' atoms]. The positions of C9 and C10 are effectively not affected by the disorder.

### Crystal data

**Table:**

C_9_H_11_N_3_S_2_	*F*(000) = 472
*M**_r_* = 225.33	*D*_x_ = 1.327 Mg m^−^^3^
Monoclinic, *P*2_1_/*c*	Mo *K*α radiation, λ = 0.71073 Å
Hall symbol: -P 2ybc	Cell parameters from 69 reflections
*a* = 6.813 (1) Å	θ = 6–14°
*b* = 17.119 (2) Å	µ = 0.44 mm^−^^1^
*c* = 9.846 (1) Å	*T* = 295 K
β = 100.88 (1)°	Prism, colourless
*V* = 1127.7 (2) Å^3^	0.47 × 0.30 × 0.16 mm
*Z* = 4	

### Data collection

**Table:**

Oxford Diffraction Xcalibur diffractometer	*R*_int_ = 0.026
Radiation source: fine-focus sealed tube	θ_max_ = 27.6°, θ_min_ = 3.9°
graphite	*h* = −8→8
ω–2θ scans	*k* = 0→22
2735 measured reflections	*l* = 0→12
2592 independent reflections	3 standard reflections every 100 reflections
1130 reflections with *I* > 2σ(*I*)	intensity decay: 0.1%

### Refinement

**Table:**

Refinement on *F*^2^	Primary atom site location: structure-invariant direct methods
Least-squares matrix: full	Secondary atom site location: difference Fourier map
*R*[*F*^2^ > 2σ(*F*^2^)] = 0.052	Hydrogen site location: inferred from neighbouring sites
*wR*(*F*^2^) = 0.111	H-atom parameters constrained
*S* = 0.98	*w* = 1/[σ^2^(*F*_o_^2^) + (0.0395*P*)^2^ + 0.2245*P*] where *P* = (*F*_o_^2^ + 2*F*_c_^2^)/3
2592 reflections	(Δ/σ)_max_ = 0.002
147 parameters	Δρ_max_ = 0.16 e Å^−^^3^
0 restraints	Δρ_min_ = −0.16 e Å^−^^3^

### Special details

**Table:**

Geometry. All e.s.d.'s (except the e.s.d. in the dihedral angle between two l.s. planes) are estimated using the full covariance matrix. The cell e.s.d.'s are taken into account individually in the estimation of e.s.d.'s in distances, angles and torsion angles; correlations between e.s.d.'s in cell parameters are only used when they are defined by crystal symmetry. An approximate (isotropic) treatment of cell e.s.d.'s is used for estimating e.s.d.'s involving l.s. planes.
Refinement. Refinement of *F*^2^ against ALL reflections. The weighted *R*-factor *wR* and goodness of fit *S* are based on *F*^2^, conventional *R*-factors *R* are based on *F*, with *F* set to zero for negative *F*^2^. The threshold expression of *F*^2^ > σ(*F*^2^) is used only for calculating *R*-factors(gt) *etc*. and is not relevant to the choice of reflections for refinement. *R*-factors based on *F*^2^ are statistically about twice as large as those based on *F*, and *R*- factors based on ALL data will be even larger.

### Fractional atomic coordinates and isotropic or equivalent isotropic displacement parameters (Å^2^)

**Table:**

	*x*	*y*	*z*	*U*_iso_*/*U*_eq_	Occ. (<1)
N1	0.2725 (3)	0.49647 (12)	0.6098 (2)	0.0563 (6)	
H1	0.1684	0.4675	0.5868	0.068*	
N2	0.4538 (3)	0.46832 (13)	0.6760 (2)	0.0598 (6)	
C3	0.5669 (4)	0.52970 (16)	0.6930 (3)	0.0547 (7)	
N4	0.4644 (3)	0.59498 (12)	0.6394 (2)	0.0504 (5)	
C5	0.2730 (3)	0.57271 (16)	0.5846 (2)	0.0501 (6)	
S1	0.08621 (10)	0.62899 (4)	0.50362 (7)	0.0654 (3)	
C6	0.7818 (4)	0.52769 (17)	0.7589 (3)	0.0703 (8)	
H6A	0.8601	0.5469	0.6933	0.084*	
H6B	0.8205	0.4739	0.7799	0.084*	
S2	0.7156 (9)	0.5621 (3)	1.0253 (6)	0.0683 (11)	0.538 (6)
C8	0.982 (3)	0.6272 (12)	0.921 (2)	0.123 (10)	0.538 (6)
H8	1.0760	0.6381	0.8662	0.148*	0.538 (6)
C8'	0.757 (3)	0.5727 (13)	1.003 (2)	0.079 (9)	0.462 (6)
H8'	0.6549	0.5383	1.0138	0.094*	0.462 (6)
S2'	1.0016 (7)	0.6482 (5)	0.9087 (7)	0.0949 (14)	0.462 (6)
C7	0.8316 (5)	0.57473 (19)	0.8885 (3)	0.0588 (8)	
C9	0.9766 (6)	0.6655 (2)	1.0589 (5)	0.1017 (13)	
H9	1.0536	0.7077	1.0974	0.122*	
C10	0.8435 (6)	0.6283 (2)	1.1134 (3)	0.0885 (10)	
H10	0.8241	0.6403	1.2020	0.106*	
C11	0.5372 (4)	0.67518 (16)	0.6362 (3)	0.0670 (8)	
H11A	0.4782	0.6990	0.5485	0.080*	
H11B	0.6810	0.6744	0.6425	0.080*	
C12	0.4875 (5)	0.72417 (17)	0.7527 (3)	0.0815 (9)	
H12A	0.5369	0.7763	0.7462	0.098*	
H12B	0.5488	0.7017	0.8397	0.098*	
H12C	0.3452	0.7257	0.7463	0.098*	

### Atomic displacement parameters (Å^2^)

**Table:**

	*U*^11^	*U*^22^	*U*^33^	*U*^12^	*U*^13^	*U*^23^
N1	0.0502 (12)	0.0529 (14)	0.0614 (14)	−0.0035 (10)	−0.0008 (10)	−0.0031 (11)
N2	0.0522 (13)	0.0619 (14)	0.0620 (15)	0.0057 (11)	0.0022 (11)	−0.0059 (12)
C3	0.0486 (15)	0.0655 (17)	0.0495 (16)	0.0044 (14)	0.0081 (12)	−0.0053 (15)
N4	0.0448 (12)	0.0561 (13)	0.0489 (12)	−0.0051 (11)	0.0056 (9)	−0.0040 (10)
C5	0.0512 (15)	0.0537 (16)	0.0441 (14)	−0.0029 (12)	0.0056 (12)	−0.0055 (13)
S1	0.0578 (4)	0.0571 (4)	0.0739 (5)	−0.0009 (3)	−0.0067 (3)	0.0003 (4)
C6	0.0456 (16)	0.088 (2)	0.075 (2)	0.0055 (15)	0.0052 (14)	−0.0120 (17)
S2	0.070 (2)	0.0744 (16)	0.0589 (15)	−0.0131 (15)	0.0081 (15)	−0.0015 (13)
C8	0.159 (19)	0.120 (15)	0.102 (10)	0.015 (11)	0.056 (10)	0.031 (9)
C8'	0.057 (9)	0.096 (10)	0.084 (15)	−0.022 (6)	0.015 (6)	0.018 (7)
S2'	0.0717 (17)	0.110 (3)	0.097 (3)	−0.0346 (17)	−0.0010 (15)	0.010 (2)
C7	0.0398 (15)	0.0610 (19)	0.071 (2)	−0.0056 (14)	−0.0015 (15)	0.0057 (17)
C9	0.104 (3)	0.072 (2)	0.109 (3)	−0.029 (2)	−0.033 (2)	0.008 (2)
C10	0.116 (3)	0.081 (2)	0.061 (2)	0.006 (2)	−0.002 (2)	−0.002 (2)
C11	0.0566 (17)	0.0687 (19)	0.0731 (19)	−0.0165 (14)	0.0055 (14)	0.0078 (17)
C12	0.079 (2)	0.0605 (18)	0.098 (2)	−0.0082 (15)	−0.0005 (17)	−0.0105 (18)

### Geometric parameters (Å, °)

**Table:**

N1—C5	1.329 (3)	C8—H8	0.9300
N1—N2	1.370 (3)	C8'—C7	1.32 (2)
N1—H1	0.8600	C8'—C10	1.48 (2)
N2—C3	1.295 (3)	C8'—H8'	0.9300
C3—N4	1.370 (3)	S2'—C9	1.549 (10)
C3—C6	1.485 (3)	S2'—C7	1.695 (6)
N4—C5	1.368 (3)	C9—C10	1.304 (5)
N4—C11	1.462 (3)	C9—H9	0.9300
C5—S1	1.673 (2)	C10—H10	0.9300
C6—C7	1.493 (4)	C11—C12	1.510 (4)
C6—H6A	0.9700	C11—H11A	0.9700
C6—H6B	0.9700	C11—H11B	0.9700
S2—C10	1.584 (7)	C12—H12A	0.9600
S2—C7	1.699 (6)	C12—H12B	0.9600
C8—C7	1.355 (17)	C12—H12C	0.9600
C8—C9	1.51 (2)		
			
C5—N1—N2	113.6 (2)	C8—C7—C6	126.8 (11)
C5—N1—H1	123.2	C8'—C7—S2'	106.5 (10)
N2—N1—H1	123.2	C6—C7—S2'	122.7 (4)
C3—N2—N1	103.7 (2)	C8—C7—S2	110.0 (11)
N2—C3—N4	111.4 (2)	C6—C7—S2	123.0 (3)
N2—C3—C6	123.4 (2)	S2'—C7—S2	114.3 (4)
N4—C3—C6	125.2 (3)	C10—C9—C8	107.1 (7)
C5—N4—C3	107.7 (2)	C10—C9—S2'	120.6 (4)
C5—N4—C11	123.7 (2)	C10—C9—H9	126.5
C3—N4—C11	128.6 (2)	C8—C9—H9	126.5
N1—C5—N4	103.6 (2)	S2'—C9—H9	112.5
N1—C5—S1	128.8 (2)	C9—C10—C8'	103.1 (8)
N4—C5—S1	127.6 (2)	C9—C10—S2	118.6 (4)
C3—C6—C7	114.1 (2)	C9—C10—H10	120.7
C3—C6—H6A	108.7	C8'—C10—H10	136.2
C7—C6—H6A	108.7	S2—C10—H10	120.7
C3—C6—H6B	108.7	N4—C11—C12	112.3 (2)
C7—C6—H6B	108.7	N4—C11—H11A	109.1
H6A—C6—H6B	107.6	C12—C11—H11A	109.1
C10—S2—C7	93.0 (4)	N4—C11—H11B	109.1
C7—C8—C9	110.7 (14)	C12—C11—H11B	109.1
C7—C8—H8	124.7	H11A—C11—H11B	107.9
C9—C8—H8	124.7	C11—C12—H12A	109.5
C7—C8'—C10	116.3 (13)	C11—C12—H12B	109.5
C7—C8'—H8'	121.9	H12A—C12—H12B	109.5
C10—C8'—H8'	121.9	C11—C12—H12C	109.5
C9—S2'—C7	93.4 (4)	H12A—C12—H12C	109.5
C8'—C7—C8	102.3 (15)	H12B—C12—H12C	109.5
C8'—C7—C6	130.7 (10)		
			
C5—N1—N2—C3	0.8 (3)	C3—C6—C7—S2'	−122.1 (4)
N1—N2—C3—N4	−0.4 (3)	C3—C6—C7—S2	54.9 (4)
N1—N2—C3—C6	−178.8 (2)	C9—S2'—C7—C8'	3.9 (11)
N2—C3—N4—C5	−0.1 (3)	C9—S2'—C7—C8	−58 (9)
C6—C3—N4—C5	178.2 (2)	C9—S2'—C7—C6	−178.6 (3)
N2—C3—N4—C11	−179.4 (2)	C9—S2'—C7—S2	4.1 (5)
C6—C3—N4—C11	−1.0 (4)	C10—S2—C7—C8'	−2(9)
N2—N1—C5—N4	−0.9 (3)	C10—S2—C7—C8	5.2 (10)
N2—N1—C5—S1	178.5 (2)	C10—S2—C7—C6	179.8 (2)
C3—N4—C5—N1	0.6 (3)	C10—S2—C7—S2'	−2.9 (4)
C11—N4—C5—N1	179.9 (2)	C7—C8—C9—C10	8.5 (15)
C3—N4—C5—S1	−178.8 (2)	C7—C8—C9—S2'	−145 (4)
C11—N4—C5—S1	0.5 (3)	C7—S2'—C9—C10	−4.2 (5)
N2—C3—C6—C7	−117.9 (3)	C7—S2'—C9—C8	25 (3)
N4—C3—C6—C7	63.9 (4)	C8—C9—C10—C8'	−5.0 (13)
C10—C8'—C7—C8	5(2)	S2'—C9—C10—C8'	2.8 (11)
C10—C8'—C7—C6	179.6 (7)	C8—C9—C10—S2	−4.9 (9)
C10—C8'—C7—S2'	−3.2 (19)	S2'—C9—C10—S2	2.9 (6)
C10—C8'—C7—S2	178 (11)	C7—C8'—C10—C9	0.6 (19)
C9—C8—C7—C8'	−7.5 (18)	C7—C8'—C10—S2	−179 (6)
C9—C8—C7—C6	177.2 (6)	C7—S2—C10—C9	0.2 (4)
C9—C8—C7—S2'	113 (9)	C7—S2—C10—C8'	1(4)
C9—C8—C7—S2	−8.4 (15)	C5—N4—C11—C12	83.3 (3)
C3—C6—C7—C8'	54.7 (14)	C3—N4—C11—C12	−97.5 (3)
C3—C6—C7—C8	−131.4 (11)		

### Hydrogen-bond geometry (Å, °)

**Table:**

*D*—H···*A*	*D*—H	H···*A*	*D*···*A*	*D*—H···*A*
N1—H1···S1^i^	0.86	2.44	3.287 (3)	169
C6—H6a···S1^ii^	0.97	2.99	3.949 (4)	172
C9—H9···S1^iii^	0.93	2.97	3.659 (4)	132
C8'—H8'···S2^iv^	0.93	3.02	3.928 (7)	166

Symmetry codes: (i) −*x*, −*y*+1, −*z*+1; (ii) *x*+1, *y*, *z*; (iii) *x*+1, −*y*+3/2, *z*+1/2; (iv) −*x*+1, −*y*+1, −*z*+2.
